# Use of a Modified Spatial-Context Memory Test to Detect Amnestic Mild Cognitive Impairment

**DOI:** 10.1371/journal.pone.0057030

**Published:** 2013-02-28

**Authors:** Hsuan-Min Wang, Chien-Ming Yang, Wan-Chin Kuo, Chin-Chang Huang, Hung-Chou Kuo

**Affiliations:** 1 Department of Neurology, Chang Gung Memorial Hospital and University Medical College, Linkou Medical Center, Taipei, Taiwan; 2 Department of Psychology, National Chengchi University, Taipei, Taiwan; 3 Department of Psychiatry, Chang Gung Memorial Hospital, Chiayi, Taiwan; Rush University, United States of America

## Abstract

In this study we sought to differentiate participants with amnestic mild cognitive impairment (a-MCI) from those with mild dementia of Alzheimer’s type (m-DAT) and normal controls by modifying an existing test of spatial context memory (SCMT) designed so as to evaluate the function of brain regions affected in early m-DAT. We found that participants with a-MCI had better total scores on our modified SCMT than those with m-DAT. Furthermore, the locational memory subtest was able to discriminate between those with a-MCI and m-DAT. Additionally, compared with other screening tests, our spatial context memory test showed high sensitivity and specificity in discerning those with a-MCI from the normal population but, was relatively ineffective in discriminating a-MCI patients from those with m-DAT. We conclude that our modified test of SCMT is an effective tool for discriminating a-MCI from m-DAT and does so by detecting differences in locational memory.

## Introduction

Amnestic mild cognitive impairment (a-MCI) has many neuropathological features implicating it as a transitional state from normal cognition to mild dementia of Alzheimer’s type (m-DAT) [1]. However, at present there is no powerful tool for early detection of a-MCI that can discriminate between a-MCI and m-DAT. However, previous research has shown that episodic memory is impaired during early stages of a-MCI, presenting as an inability to retrieve the spatial context [2] of an event as well as errors in spatial navigation [3] which are usually linked to pathological changes within the hippocampus and associated structures. Furthermore, a-MCI has pathology similar to that of m-DAT, which involves damage to three main brain areas: the hippocampal complex, cingulate gyrus, and tempo-parietal region [4] As the perirhinal cortex and parahippocampus are responsible for feature encoding and the hippocampus is primarily involved with spatial location memory representation [3], [5]–[7], we designed our test to distinguish between these functions.

Various tests are available for diagnosing various conditions, including a-MCI and m-DAT [8]. However, many of these tests have been found to have varying levels of differential diagnostic power. For example, the widely used Clock Drawing Test part D is unable to differentiate a-MCI patients from normal controls [9], while the Visual Association Memory Test (VAMT), designed to test for antegrade amnesia has been found to be effective in detecting early Alzheimer’s disease (AD) [10]. Also, deficits in episodic memory as determined by a battery of neuropsychological tests have been shown to predict later conversion from a-MCI to m-DAT [8].

In view of the spatial context memory function of the hippocampal complex region, we hypothesized that the item and scene features association memory, but not spatial location memory would be impaired in a-MCI while spatial location memory would be affected in early AD. We feel if the different stages could be categorized by performances on a spatial context memory test of our own design; it could be utilized in clinical settings to assist the diagnosis of a-MCI.

## Materials and Methods

### Clinical Evaluation and Neuropsychological Tests

Participants (*N* = 60) in this study were recruited through the dementia clinic at Chang Gung Memorial Hospital patients and grouped as a-MCI (n = 30) or m-DAT (n = 30). Informed consent was obtained from each participant in accordance with protocol approved by Chang Gung Memorial Hospital (IRB 97-0638B and IRB: 99-1321C). The study was approved by the Institutional Review Board of Chang Gung Memorial Hospital. Normal controls (n = 30) were required to show no signs of cognitive impairment, be more than 55 years old and were recruited from a pool including patient’s spouses, hospital volunteers, or individuals from the surrounding community. All participants received a standard neurological examination to rule out other neurological disease, major psychiatric illness, and severe visual or hearing impairment. All participants also received blood tests to rule out other systemic diseases including whole blood counts, electrolytes, sugar levels, renal and liver function, serum vitamin B12, and folic acid levels. Serological tests for syphilis and endocrine function of both thyroid and adrenal glands were also performed. Furthermore, all patients underwent brain computer tomogram (CT) or magnetic resonance imaging (MRI) to rule out the possibility of other brain lesions. All individuals were identified as normal controls, a-MCI, or m-DAT using the aforementioned clinical data, family information, and neuropsychological tests. The demographic data of the patients are shown in Table 1.

**Table 1:** Patient Demographics and Clinical Dementia Rating scores.

	NC (n = 30)	a-MCI (n = 30)	m-DAT (n = 30)	*P*-value
Age (years)	71.00±6.99	73.70±7.40	75.17±8.16	0.101
Gender, n (%)				
Male	16 (46.7)	16 (46.7)	15 (50.0)	0.956
Female	14 (53.3)	14 (53.3)	15 (50.0)	
Education (yeas)	8.93±4.1	8.43±3.42	8.13±3.41	0.696
Global CDR	0	0.47±013†	0.87±0.22† ‡	<0.001*
CDR-SOB score	0.02±0.09	1.38±0.98†	4.63±1.42† ‡	<0.001*

*P*-values are based on ANOVA test.

* Indicates a significant difference, *P*<0.05.

† Indicates a statistically significant difference between the indicated group and the normal control group.

‡ Indicates a statistically significant difference between the a-MCI and mild m-DAT group.

Pair-wise multiple comparisons between groups were determined using Bonferroni test with α = 0.017 (0.05/3) adjustment.

NC: normal control; a-MCI: amnestic mild cognitive impairment; m-DAT: mild dementia of Alzheimer’s type; CDR: clinical dementia rating scale; SOB: sum of boxes.

Patients were diagnosed with potential m-DAT according to the criteria of the Diagnostic and Statistical Manual of Mental Disorders, 4th edition (DSM-IV) [11] and National Institute of Neurological and Communicative Disorders and Stroke/Alzheimer’s Disease and Related Disorders Association (NINCDS-ADRDA) [12]. Disease severity was graded by using Clinical Dementia Rating (CDR) and Mini-Mental Status Examination (MMSE) [13] to determine cognitive function. A diagnosis of m-DAT was determined by a score of 1 on the CDR. A diagnosis of a-MCI was defined by subjective memory impairment and having significant deficits in memory (ie. <1.5 standard deviations (SD)) below the age and education control mean on the tests of delayed recall) while other neuropsychological functions remained within normal parameters (ie. within 0.5 SD of appropriate controls) [1]. A diagnosis of a-MCI also required that the patient showed delayed recall, retention, and recognition on the Word List Memory subtest of more than 1.5 SD below the age and education adjusted norm while also scoring within 0.5 SD of age and education norms on the MMSE and Boston Naming Test of Consortium to Establish Registry for Alzheimer’s Disease (CERAD) [14]. As the Word List Memory and Boston Naming Tests were time consuming and mainly used to assist in distinguishing the a-MCI and NC groups, they were not performed on the probable m-DAT group. Participants with a-MCI were also required to show that they retained intact and independent activities of daily living that did not meet the criteria of DSM-IV [11] and NINCDS-ADRDA [12] for possible or probable AD. Most of participants with m-DAT and a-MCI who could tolerate long visits completed the Behavioral Pathology in Alzheimer’s Disease Scale (BEHAVE-AD), Cognitive Abilities Screening Instrument (CASI), and Alzheimer’s Disease Assessment Scale Cognitive Subscale (ADAS-Cog) [15]. The neuropsychological tools utilized for diagnosis in this study are listed in Table 2. In addition, a modified SCMT consisting of 3 separate subtests was performed to evaluate its effectiveness in distinguishing a-MCI from m-DAT, which is described below.

**Table 2:** Standard Neuropsychological Test scores.

Test	NC group (n = 30)	a-MCI group (n = 30)	m-DAT group (n = 30)	*P*-value
***Neuropsychological tests***				
MMSE^1^	28.06±1.65	25.86±2.19†	17.86±3.25† ‡	<0.001*
*CERAD*				
Category fluency test^2^	13.08±3.06	10.09±1.56	–	<0.001*
Boston naming test ^2a^	4.80±0.61	4.06±1.01	–	<0.001*
Retention of WLM^2^	89.57±9.98	61.33±21.51	–	<0.001*
Registration of WLM^2b^	8.33±1.21	6.96±1.47	–	<0.001*
Delayed recall of WLM^ 2a^	3.43±0.97	1.56±0.97	–	<0.001*
Recognition of WLM^ 2a^	4.20±0.88	2.46±1.50	–	<0.001*
Retention of visual construction^1^	82.59±16.85	43.07±24.62†	10.26±17.74† ‡	<0.001*
TMT A time^1^	68.00±25.11	108.86±38.49†	149.23±80.13† ‡	<0.001*
TMT B time^2^	167.54±83.69	233.25±101.72	–	0.046*
Clock Drawing Test^1^	14.70±1.23	12.56±2.77	9.93±4.65† ‡	<0.001*
VAMT 1^1^	4.93±0.94	3.55±1.73†	0.96±1.31† ‡	<0.001*
VAMT 2^1^	5.73±0.63	4.37±1.77†	1.29±1.63† ‡	<0.001*
Total score of VAMT^1^	10.66±1.37	7.92±3.41†	2.25±2.89† ‡	<0.001*

*P*-values are based on ^1^ANOVA test and ^2^ independent two sample test.

NC: normal control; a-MCI: amnestic mild cognitive impairment; m-DAT: mild dementia of Alzheimer’s type.

† Indicates a statistically significant difference between the indicated group and the normal control group.

‡ Indicates a statistically significant difference between the a-MCI and m-DAT group.

CERAD: Consortium to Establish Registry for Alzheimer’s Disease; MMSE: Mini-mental status examination; WLM: word list memory test; TMT: trail making test; VAMT: visual association memory test;-: not available

a score adjusted to account for age and education bias;

b highest score from 3 trials;

* Indicates a significant difference, *P*<0.05.

### Modified Spatial-Context Memory Test

The modified SCMT includes 3 subtests of spatial location, event-place association, and place-object association memory, and was modified from the works of Burgess and colleagues [5], [6] in accordance with previous functional neuroimaging studies [7], [16]. For this study, the SCMT was given to all subjects to evaluate spatial context memory. The 3 subtests were: 1) a spatial location memory subtest that mainly tests hippocampal function, 2) an event-place association test, and 3) a place-object association memory subtest, which is used to assess the functionality of the bi-directional connection between perirhinal and parahippocampal regions [5], [6]. During all tests, the subjects were requested to sit 45 cm from a non-reflective computer screen with a clear view of the stimulus. The stimulation test was carried out using the computer and the completed testing time was usually between 15 to 20 minutes. The three subtests were designed as follows:

#### Spatial location memory subtest

**Figure 1:**
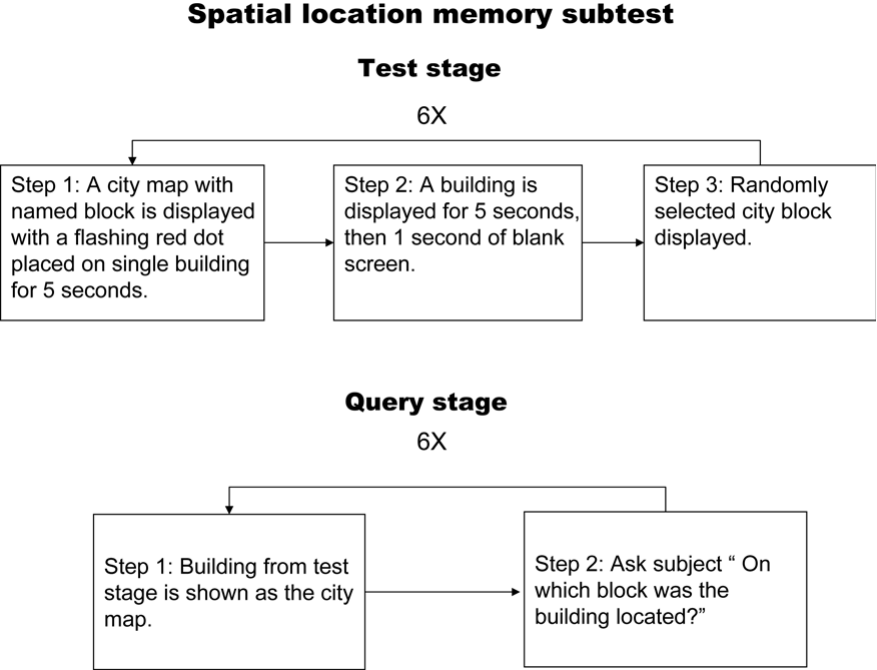
Flow chart illustrating the spatial context memory test.

A flow chart illustrating the spatial location memory test is shown in Figure 1. Briefly, during the appearance the stimulus, a city map would be presented with blocks of buildings (identified by name) arranged around the map. A flashing red dot would appear on a particular block in the map and after the red dot flashed for 5 seconds, the screen would be switched to show photos of this building. This was followed by one second of blank screen, and then another randomly chosen block was used for a total of 6 different locations. After all 6 different stimulations were shown, there were 5 seconds of blank screen, and then the inquiry period would begin. This involved presenting photos of a specific building with the subject asked to identify its location on the map. One point was given for every correct answer with a highest possible score of 6.

#### Event-place association memory subtest

**Figure 2:**
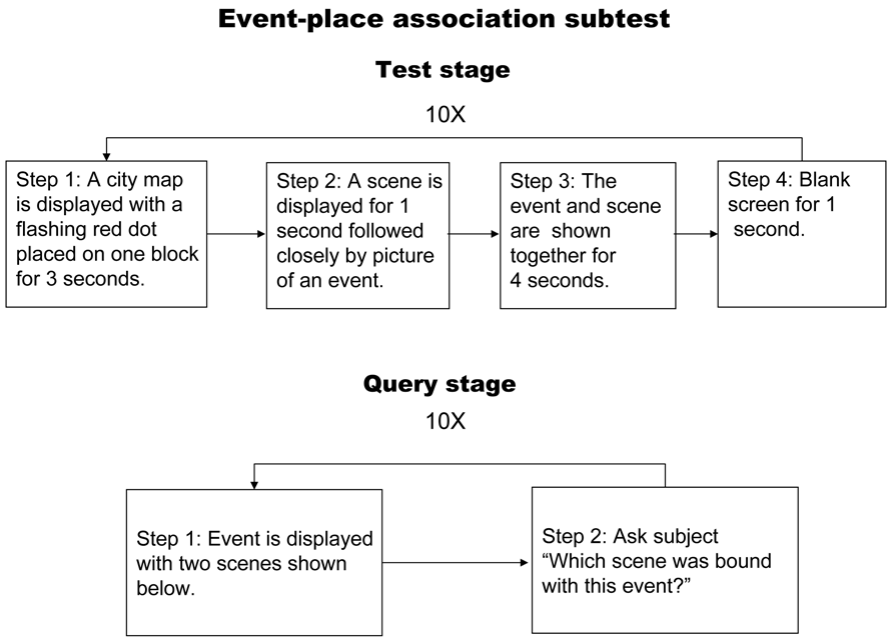
Flow chart illustrating the event-place memory test.

A flow chart describing the event-place association memory subset is shown in Figure 2. Briefly, as in the previous test, a city map would be first presented, and a flashing red dot appeared on a specific block in the map so as to minimize any bias between tests resulting from attention differences. This red dot flashed for 3 seconds and then the screen was switched to show a black-and white photo of a scene for one second, after which an event would tightly follow. Then the scene and the event would appear together for 4 seconds, followed by a blank screen for one second before continuing to another set. This was repeated for a total of 10 sets of scene and event associations, after which there would be 5 seconds of blank screen followed by the inquiry. During the inquiry period, the events were shown and the subject was asked which one of the two scenes below it was associated with. There were a total of 10 questions with 1 point given for each correct answer.

#### Place-object association memory subtest

**Figure 3:**
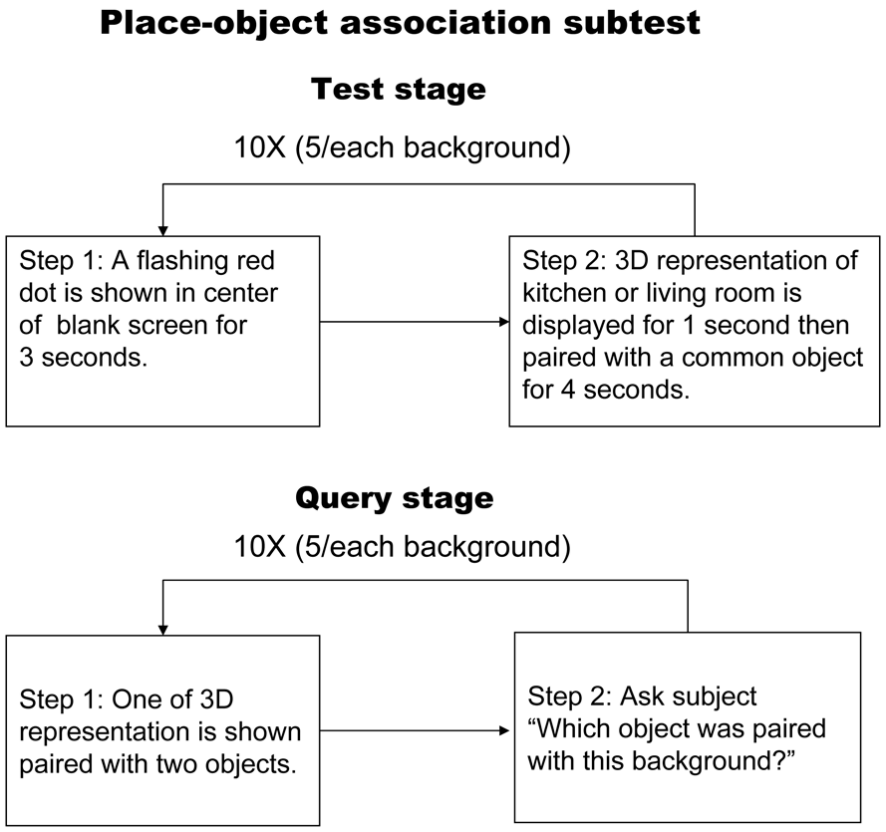
Flow chart illustrating the place-object.

A flow chart describing the place-object association memory subset is shown in Figure 3. Briefly, two different backgrounds (a 3D representation of a living room or kitchen) would randomly appear, then a red dot would flash for 3 seconds in the center of the screen, and then the screen would be switched to show the background for one second. This was quickly followed by a presentation of the background in combination with a daily life good or similar object for 4 seconds, followed by a blank screen for one second before continuing to another set. There were a total of 10 sets of combined stimuli with each background eventually being paired with 5 different objects. During the inquiry period, one of the backgrounds would be presented followed by 2 objects located below it. The patient was then asked to identify which one was paired with the presented background. This was repeated for a total of 10 questions with each correct answer given 1 point. The total score was the sum of all three subtests and ranged from 0 to 26.

### Statistical Analysis

Differences in patient demographics and neuropsychological test results between the 3 groups were determined using a one-way ANOVA. If a significant difference between groups was found, post-hoc multiple comparisons of means were performed using the Bonferroni procedure with type-I error adjustment. To determine the difference in the scores on neuropsychological tests between normal controls and the a-MCI group, we used an independent two-sample t-test. Furthermore, to analyze the discriminative power of the different neuropsychological tests, a receiver-operating characteristic curve analysis was used to calculate the area under the curve, sensitivity, and specificity, along with the positive and negative predictive values for each individual test. All statistic assessments were two sided and *P*≤0.05 was considered statistically significant. All statistical analyses were performed using SPSS 15.0 statistics software (SPSS Inc, Chicago, IL).

## Results

Demographic characteristics and Clinical Dementia rating scores of the 3 groups are shown in Table 1. There were no significant difference in age, gender, and duration of education between the 3 groups (*P*>0.05). However, participants with m-DAT showed the highest Clinical Dementia rating scores. In addition, we found no significant difference between measures of community, home & hobbies, and personal care between normal controls and a-MCI groups. As the results of neuropsychological tests are vulnerable to the impact to age and education, we used an adjusted norm. If the tests lacked an adjusted norm, we used the covariance to decrease any age and education interference. In addition, while the CERAD battery test was used to distinguish the a-MCI from normal controls, it was not used to assess the m-DAT Group.

Table 2 shows the results of standard neuropsychological testing across all 3 groups. There were significant differences in MMSE, retention of visual construction, tail making test, clock drawing test part D, and visual association memory test. Post-hoc analysis revealed that among 3 groups, the m-DAT group had significantly lower scores on the MMSE, retention of visual construction, clock drawing test part D, and visual association memory test. The a-MCI group had significantly lower scores on the rest neuropsychological tests than normal control group (all *P*<0.05).

**Figure 4:**
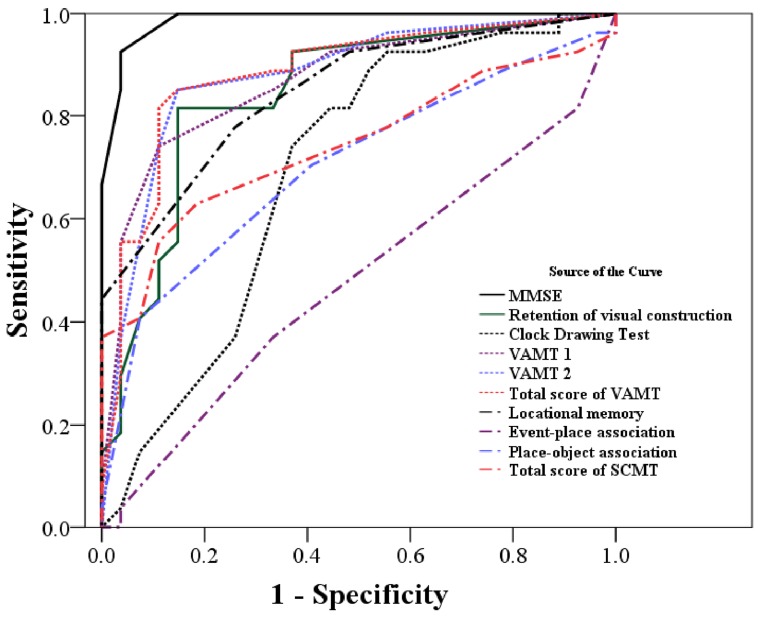
Receiver operating characteristic test showing the ability of each neuropsychological test to discriminate between the amnestic mild cognitive impairment and mild dementia of Alzheimer’s type groups. The MMSE shows the highest discriminative power of the tests used in this study. MMSE: Mini-mental status examination; VAMT: visual association memory test; TMT: trail making test; CERAD: Consortium to Establish Registry for Alzheimer’s disease; SCMT: Spatial Context Memory Test.

The ROCs for the a-MCI and m-DAT groups on each neuropsychological test are shown in Figure 4. This analysis found that among all tests studied, the MMSE was best in differentially diagnosing participants with a-MCI from those with mild m-DAT (93% sensitivity and 96% specificity; Table 3).

**Table 3:** Area under the curve for Standard Neuropsychological test scores.

	NC vs a-MCI AUC (95% CI)	*P*-value	a-MCI vs m-DAT AUC (95% CI)	*P*-value
MMSE	0.79 (0.68, 091)	<0.001*	0.99 (0.97, 1.00)	<0.001*
Retention of visual construction	0.93 (0.86, 0.99)	<0.001*	0.84 (0.74, 0.95)	<0.001*
Clock Drawing Test	0.82 (0.70, 0.93)	<0.001*	0.69 (0.55, 0.84)	0.015*
VAMT 1	0.74 (0.61, 0.87)	0.002*	0.87 (0.77, 0.97)	<0.001*
VAMT 2	0.75 (0.62, 0.88)	0.001*	0.88 (0.78, 0.98)	<0.001*
VAMT (Total)	0.77 (0.64, 0.89)	0.001*	0.88 (0.79, 0.98)	<0.001*

* Statistically significant.

NC: normal control; a-MCI: amnestic mild cognitive impairment; m-DAT: mild dementia of Alzheimer’s type; MMSE: Mini-mental status examination; VAMT: visual association memory test.

Table 4 shows the results of the modified SCMT location memory, event-place association, and place-object association subtests, as well as total SCMT scores among the 3 groups (all *P*<0.001). Post-hoc analysis revealed that among the 3 groups, the m-DAT group had the lowest scores in both the locational memory subtest and total score (both *P*<0.05). The a-MCI and m-DAT groups had comparable scores in both event-place association subtest and place-object association subtest, which were significantly lower than the corresponding scores in the normal control group.

**Table 4:** Spatial Context Memory Test subtest scores.

SCMT subtest	NC (n = 30)	a-MCI (n = 30)	m-DAT (n = 30)	*P*-value
Locational memory subtest	4.46±1.04	2.26±1.22†	0.73±0.82† ‡	<0.001*
Event-place association subtest	7.20±0.85	3.83±1.17†	3.93±1.08†	<0.001*
Place-object association subtest	7.60±1.86	5.26±1.72†	4.30±1.02†	<0.001*
Total score	19.26±2.94	11.36±3.02†	8.96±1.88† ‡	<0.001*

* Statistically significant;

† Indicates a statistically significant difference between the indicated group and the normal control group.

‡ Indicates a statistically significant within group difference between the a-MCI and m-DAT group. NC: normal control; a-MCI: amnestic mild cognitive impairment; m-DAT: mild dementia of Alzheimer’s type; SCMT: Spatial Context Memory Test.

**Figure 5:**
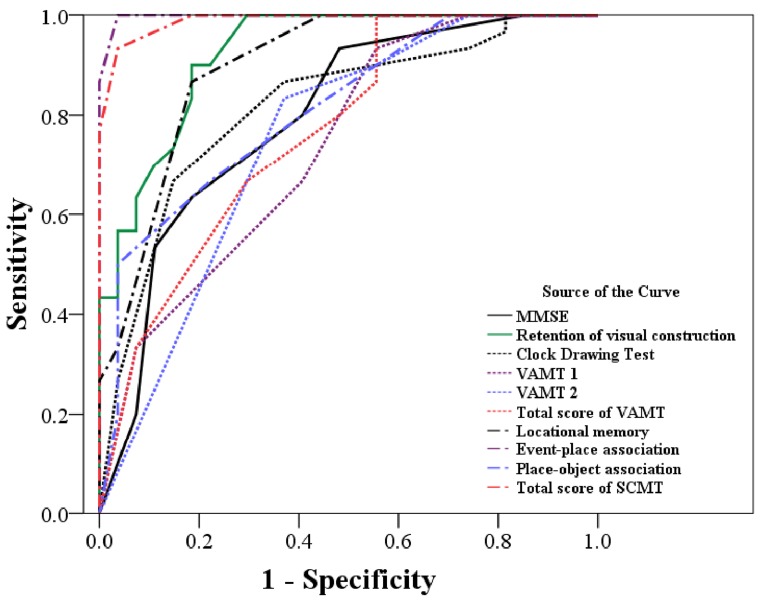
Receiver-operating characteristic curve showing the ability of each neuropsychological test to discriminate between normal controls and amnestic mild cognitive impairment patients. The event-place association and the total spatial context memory test score show higher discriminative power than the other tests used in this study. MMSE: Mini-mental status examination; VAMT: visual association memory test; TMT: trail making test; CERAD: Consortium to Establish Registry for Alzheimer’s disease; SCMT: Spatial Context Memory Test.

Figure 5 shows the receiver-operating curve (ROC) of the normal control and a-MCI groups for all neuropsychological tests. The AUC indicates that event-place association memory subtest was the most powerful of all tests in discriminating normal controls from the a-MCI group, reaching optimal results with cut point 5 (97% sensitivity, 100% specificity, 100% positive predictive values, and 96% negative predictive value). The SCMT total score also showed significant discrimination power, reaching the optimal results with cut point 15 (97% sensitivity, 93% specificity, 93% positive predictive values, and 96% negative predictive value; Table 5). Therefore, while the total SCMT score is comparatively better than the MMSE in detecting the a-MCI from normal controls it is not capable of distinguishing a-MCI from m-DAT (Table 3 and Table 5).

**Table 5:** Area under the curve for Spatial Context Memory Test subtest scores.

	NC vs a-MCI		a-MCI group vs. m-DAT	
	AUC (95% CI)	*P*-value	AUC (95% C I)	*P*-value
***SCMT***				
Locational memory	0.90 (0.82, 0.98)	<0.001*	0.85 (0.75, 0.95)	<0.001*
Event-place association	0.99 (0.99, 1.00)	<0.001*	0.48 (0.32, 0.64)	0.789
Place-object association	0.81 (0.70, 0.92)	<0.001*	0.70 (0.56, 0.85)	0.010*
Total score	0.99 (0.97, 1.00)	<0.001*	0.74 (0.61, 0.88)	0.002*

* Statistically significant.

AUC: area under the curve; NC: normal control; a-MCI: amnestic mild cognitive impairment; m-DAT: mild dementia of Alzheimer’s type; SCMT: Spatial Context Memory Test.

## Discussion

Our study found that while there are some differences between the scores of m-DAT and a-MCI patients on various neuropsychological tests, the modified SCMT was most effective at distinguishing between the a-MCI group and normal controls. Furthermore, we found that by modifying the SCMT based upon the underlying physiological damage associated with these disorders, we were able to more accurately differentiate between a-MCI and m-DAT. We conclude that tests involving spatial memory, especially our modified SCMT, can be used as diagnostic tools to better identify a-MCI. Our results support our hypothesis and suggest that the initial neuropathological changes within the parahippocampus and entorhinal regions found with a-MCI can produce spatial context memory deficits similar to those found in m-DAT.

Our research also used tree model analysis for common clinical screening tests, including MMSE, VAMT, retention of visual construction, Boston naming test, category fluency test, trail making test, and clock drawing test with the purpose of determining which tests could be used to differentially diagnose normal controls and participants with a-MCI. Interestingly, while the VAMT can differentiate early AD from vascular dementia [17], we did not find it effective in differentiating a-MCI from m-DAT. Besides SCMT, our results (data not shown) indicated that combining the retention of visual construction and trail-making tests was the most powerful test in distinguishing normal controls from the a-MCI group, while MMSE is the most powerful test for distinguishing the a-MCI and m-DAT groups.

As stated above, we believe that the results of these tests suggest the underlying neuropathologies of a-MCI and m-DAT. In AD patients, changes in cerebral blood perfusion generally begins in the hippocampal complex region, with neurofibrillary tangles also appearing early in the entorhinal region [18], [19]. Furthermore, changes in cerebral blood perfusion are also found in the parahippocampus of a-MCI patients [20], [21], an area responsible for spatial related information [5]. During the preclinical stage of AD, neurofibrillary tangles start to appear in the hippocampal complex region, particularly in parahippocampus and entorhinal regions responsible for retrieving spatial background information and incident features [5], areas also affected in m-DAT [4]. Therefore, we hypothesized that normal controls would perform better on tests of spatial context than a-MCI and m-DAT groups, with differences also apparent between the a-MCI and m-DAT groups.

We found that our modified SCMT has greater sensitivity, specificity, as well as having both positive and negative predictive values compared to other screening tests in discriminating a-MCI from normal aging. Furthermore, we found that in addition to the sum of the spatial location memory and event-place association memory subtests, total SCMT scores were able to consistently distinguish normal controls from the a-MCI group. However, while previous studies have not been able to differentiate between a-MCI and m-DAT [22], [23], the locational memory subscore and total score (and to a lesser extent place-object association subtest scores) were capable of distinguishing a-MCI and m-DAT. Furthermore, the inability of the clock-drawing test to differentiate the a-MCI group from normal controls confirms a recent report with similar results [9].

Regarding place-object association memory, we predicted that normal controls would perform better than both a-MCI and m-DAT groups with no apparent differences between the a-MCI and m-DAT groups. However, while we found that while normal controls performed better than the a-MCI group, the a-MCI group performed significantly better than the m-DAT group. When we further analyzed the incorrect items we found that the subjects used life experience to help choose which object and background bound would make sense. For instance, in Taiwanese culture pairing “living room” with “umbrella” would be more intuitive than pairing “kitchen” with “umbrella”, therefore subjects tended towards choosing “umbrella” with the background “living room”. In addition, these associations involved easily formed lingual meanings and are usually linked to subject’s personal experiences. As a result, this subtest may not be a pure spatial context memory test, but one that involves other cognitive abilities, such as logical reasoning ability, concept forming ability, or retrieval of personal experience, which would explain why participants with m-DAT show poorer performance than those with a-MCI.

Our study indicates the a-MCI group may have deficits in representing spatial context memory. We hypothesize that this may be linked to damage of the parahippocampus and entorhinal regions [24]. However, while we were unable to evaluate the correlation between SCMT scores and disease severity in those participants, our findings still contribute to the current literature on spatial context memory in a-MCI.

There are limitations to this study. Both a-MCI and m-DAT involve damage to the hippocampus, which is responsible for spatial context memory [5], [24]–[26]. However, in this study we are unable to determine the actual neuropathological changes in the hippocampus for participants with a-MCI, as we were limited to the subject’s clinical data, neuropsychological test results, and family information for inclusion criteria into the a-MCI group. This heterology between the subjects might cause the discrepant performance in locational memory in some participants with a-MCI. This may also be explained by studies showing that about 50% of a-MCI patients show pathology associated with m-DAT, which would make differentiation difficult [1].

From a behavioural standpoint, studies on spatial location memory in mice have found that they are able to analyze their relative position and the direction towards a destination using hippocampal integration [25]. Our study utilized two-dimensional tools in attempt to imitate the representation of a spatial map within the mind. However, a two-dimensional plane could either represent spatial location memory, or spatial context memory by using the city map as background. If the latter is true, the a-MCI group should perform worse than the normal controls group, which would require future experiments for confirmation.

This study has found that our modified SCMT is comparatively better in detecting a-MCI from normal aging individuals. However, this research is a cross sectional study and it will be necessary to follow up longitudinally. In addition, advanced imaging techniques, such as amyloid-positron emission testing or f-MRI may also help to study the relationship between neuropsychological functional tests and pathological findings in the corresponding brain region.
